# Antioxidant and Anti-Proliferative Properties of *Hagenia abyssinica* Roots and Their Potentially Active Components

**DOI:** 10.3390/antiox9020143

**Published:** 2020-02-06

**Authors:** Minxia Fan, Guilin Chen, Yongli Zhang, Lutfun Nahar, Satyajit Dey Sarker, Guangwan Hu, Mingquan Guo

**Affiliations:** 1 CAS Key Laboratory of Plant Germplasm Enhancement and Specialty Agriculture, Wuhan Botanical Garden, Chinese Academy of Sciences, Wuhan 430074, China; fanminxia14@mails.ucas.ac.cn (M.F.); glchen@wbgcas.cn (G.C.); zhangyongli@wbgcas.cn (Y.Z.); guangwanhu@wbgcas.cn (G.H.); 2 University of Chinese Academy of Sciences, Beijing 100049, China; 3 Sino-Africa Joint Research Center, Chinese Academy of Sciences, Wuhan 430074, China; 4 Innovation Academy for Drug Discovery and Development, Chinese Academy of Sciences, Shanghai 201203, China; 5 Laboratory of Growth Regulators, Institute of Experimental Botany ASCR & Palacký University, Šlechtitelů 27, 78371 Olomouc, Czech Republic; drnahar@live.co.uk; 6 School of Pharmacy and Biomolecular Sciences, Centre for Natural Products Discovery, Liverpool John Moores University, James Parsons Building, Byrom Street, Liverpool L3 3AF, UK; S.Sarker@ljmu.ac.uk

**Keywords:** *Hagenia abyssinica*, *Hagenia*, anticancer, antioxidant, anti-proliferative

## Abstract

*Hagenia abyssinica* (Bruce) J. F. Gmel. is a multipurpose dioecious tree that has been used to treat various ailments, for example, the flowers of *H. abyssinica* have been widely used as a tea to treat intestinal parasites by local residents and the roots of *H.* *abyssinica* could also be used for anticancer purposes. Antioxidant activity could be one of the most important pathways to suppress cancer and there is hardly any information available on the specific chemical components corresponding to the bioactivities of *H. abyssinica* to date. The present study intended to screen and evaluate the antioxidant and anti-proliferative properties of five different fractions from *H. abyssinica* along with their corresponding total flavonoid and phenolic contents and then further identify those compounds with the most potent antioxidant and anti-proliferative activities using high performance liquid chromatography (HPLC) coupled to mass spectrometry (MS) and nuclear magnetic resonance (NMR). The total flavonoid and phenolic content assays showed that the ethyl acetate (EA) fraction of *H. abyssinica* had higher flavonoid and phenolic levels than the other four fractions. Furthermore, the 2,2-diphenyl-1-picrylhydrazyl (DPPH) superoxide radical scavenging abilities, total antioxidant capacity (TAC) assay with 2,2′-azino-bis(3-ethylbenzthiazoline-6-sulfonic acid (ABTS), and ferric-reducing antioxidant power (FRAP) were measured to evaluate the antioxidant activities of the five fractions and some pure compounds isolated from the EA fraction, which displayed higher antioxidant properties than that of the other fractions. Caffeic acid from the EA fraction showed even stronger DPPH scavenging ability (IC_50_ 7.858 ± 0.31 µg/mL) than that of Vc (IC_50_ 8.27 ± 0.11 µg/mL) as the positive control. The anti-proliferative properties of four fractions and the ethanol extract were evaluated by the 3-(4,5)-dimethylthiahiazo (-z-y1)-3,5-di-phenytetrazoliumromide (MTT) assay and the EA fraction exhibited higher anti-proliferative activities against three cancer cell lines than that of the other fractions. Additionally, the compounds with good antioxidant activity from the EA fraction of *H. abyssinica* were screened and identified using LC-MS and NMR and were also found to possess good anti-proliferative activity. In the MTT assay, the quercetin showed the strongest dose-dependent anti-proliferative activities to colon cancer cells (HT-29) and liver cancer cells (HepG2) among all of the compounds isolated. This study provided valuable information on the synergistic antioxidant and anti-proliferative properties of *H. abyssinica*.

## 1. Introduction

*Hagenia abyssinica* (Bruce) J. F. Gmel, commonly known as ‘kosso’ and ‘African rosewood’, belongs to the monospecific genus *Hagenia* of the family Rosaceae [1,2]. The ecological distribution of this plant starts in northern Ethiopia and ends in southern Zimbabwe and also occurs in Kenya, Tanzania, Uganda, Sudan, Congo, Malawi, Burundi, and Rwanda [2,3]. *H. abyssinica* is one of the main medicinal plants used by Ethiopian rural communities to treat diarrhea, tongue infection, ulcer, and other diseases [4,5]. In particular, a small amount of flowers are boiled as tea and frequently used to treat intestinal parasites in some places in African countries [6]. Meanwhile, the kosins from the female flowers have been used to repel intestinal parasites (tapeworms) [5] and have significant cytotoxicity to mouse malignant adenoma cells [7], while the male flowers of the *H. abyssinica* have the ability to induce vomiting [8]. As a traditional abortion herbal medicine, *H. abyssinica* has some potential contraceptive effects [9]. It has also been proven to cause optic atrophy [10], but, on the other hand, it can also be used to treat eye diseases [11]. In addition, the root of *H. abyssinica* is used to treat cancer along with other medicinal plants in Kofele by local doctors [6]. However, *H. abyssinica* has been overexploited and is regarded as one of the endangered tree species due to its economic, ecological, and medicinal importance [12].

Reactive oxygen species (ROS), including oxygen and non-radicals, are a constantly generating collective during physiological process [13,14]. Imbalanced levels of ROS induces oxidative stress that causes destructive actions on cellular macromolecules, leading to various diseases [14,15]. Excessive production of ROS that destroy the antioxidant defense system can thereby oxidize the biomolecules in cells. A large number of studies have shown that ROS are closely related to the development and progression of carcinogenesis in every aspect, like cell transformation, proliferation, apoptosis, metastasis, and angiogenesis [16]. Meanwhile, the ROS are shown to cause genotoxic damage, like DNA damage [17]. Various epidemiological data suggest that antioxidants are the first line of defense to regulate important signaling transduction pathways, such as mitogen-activated protein kinases (MAPKs), phosphatidylinositide 3-kinases/ protein kinase B (PI3K/Akt), nuclear factor kB (NF-kB), and nuclear factor erythroid-2-related factor 2 (Nrf2), by repairing damaged DNA, reducing cell proliferation, metastasis, and angiogenesis, and balancing the level of proapoptotic and antiapoptotic proteins to suppress carcinogenesis initiation [16]. Hence, cancer cells, displaying unrestrained growth and division, genetic instability, and senescence evasion [18,19,20], can be effectively prevented and reversed without harming normal cells by oxidative modifications of DNA, leading to the reduction of cellular levels of ROS. At present, chemoprevention is an increasingly applied strategy to halt the development of cancer and natural secondary metabolites as active components have caused great concern because of their obvious inhibitory or preventive effects on cancer. 

The main chemical components of *H. abyssinica* are phloroglucinol derivatives, phenols, saponins, flavonoids, anthraquinones, terpenoids, alkaloids, steroids, glycosides, and tannins [21,22,23,24,25]. Numerous experimental and epidemiological studies have demonstrated that secondary metabolites, such as phenolics, flavonoids, isoflavones, flavones, anthocyanins, catechin, isocatechin, and carotenoids, were able to prevent or slow down malignancy by preventing oxidative stress [26]. Hence, we could infer that *H. abyssinica* may have potential antioxidant and anti-proliferative activities according to the structure-activity relationship of its secondary metabolites. Thus, we speculated on the possibility of using *H. abyssinica* as a source of chemoprevention agents to prevent or reverse the occurrence of cancer based on its traditional anticancer application. However, most of the previous studies on *H. abyssinica* have focused on its anthelmintic activities [27,28] and very few efforts have been put on the antioxidant and anti-proliferative properties of *H. abyssinica* to date.

Therefore, the main goal of this study was to evaluate, for the first time, the antioxidant and anti-proliferative properties of the ethanol extract and the other four extracted fractions (*n*-hexane (Hex), dichloromethane (DCM), ethyl acetate (EA), and water) of the roots of *H. abyssinica* to expand the potential pharmacological activity of this plant. In addition, the total phenolic content (TPC) and total flavonoid content (TFC) of the fractions above were also measured to reveal their correlations to the antioxidant activities of those corresponding fractions. Furthermore, the compounds with good antioxidant and anti-proliferative activity from the EA fraction of *H. abyssinica* were then isolated, established, and screened by HPLC, LC-MS, and NMR. At last, the correlations between the antioxidant properties and anti-proliferative activity were also discussed in this context.

## 2. Materials and Methods

### 2.1. Plant Materials

Dried roots of *H. abyssinica* (7.3 kg) were collected from Mount Kenya (Meru, Kenya) and the identity of this plant species was confirmed by a senior taxonomist, Professor Guangwan Hu, from the Key Laboratory of Plant Germplasm Enhancement and Specialty Agriculture of Wuhan Botanical Garden, Chinese Academy of Sciences. The specimens were stored in the herbarium of this Key Laboratory with the voucher specimen numbers (No. 20140302).

### 2.2. Chemicals and Reagents

Rutin was purchased from J&K Scientific Ltd. (Beijing, China), 2,2-diphenyl-1-picrylhdrazyl (DPPH), 2,2′-azinobis-(3-ethylbenzthiazoline-6-sulf onic acid) diammonium salt (ABTS), 2,4,6-Tri(2-pyridyl)-1,3,5-triazine (TPTZ), 6-hydroxy-2,5,7,8-tetramethylchroman-2-carboxylic acid (Trolox), Vc, butylated hydroxytoluene (BHT), and foline-phenol were purchased from Sigma-Aldrich Corp. (Shanghai, China). The purity of the related standards above was equal or greater than 99.5%. The HPLC-grade solvents, such as acetonitrile (ACN), methanol, and formic acid (FA), were obtained from TEDIA Company Inc. (Fairfield, OH, USA). All of the other solvents were purchased from Shanghai Chemical Reagent Corp. (Shanghai, China).

### 2.3. Extraction, Separation and Identification

The dried plant materials (7.3 kg) were powdered and extracted by maceration with 95% ethanol (3 times, 2 d/time) at room temperature to get ethanol extract (580 g), which was then suspended in water and extracted with *n*-hexane, dichloromethane, and ethyl acetate in sequence to obtain corresponding extracts, *n*-hexane (Hex) (A, 89.3 g), dichloromethane (DCM) (B, 39.2 g), ethyl acetate (EA) (C, 233.4 g), and a water layer. The ethanol extract and four fractions (10 g each) were taken for TFC and TPC assays and analysis.

The LC-MS analysis was conducted on a TSQ Quantum Access MAX mass spectrometer coupled with an HPLC system (Thermo Accela 600, Thermo Fisher Scientific, San Jose, CA, USA). The conditions of HPLC were set as follows: 280 nm, ultrapure water (mobile phase A) and acetonitrile (mobile phase B); 5–10% B in 0–3 min, 10% B in 3–10 min, 10–30% B in 10–80 min, and 30–95% B in 80–120 min (Waters Symmetry RP-C18 column, 4.6 × 250 mm, 3.5 m, Waters, Milford, MA, USA); 37 °C; 10 µL (injection volume); and 0.6 mL/min (flow rate). The conditions for MS were the following: negative full scan and the data-dependent mode; 120 to 1000 (*m/z*); 3 kV (spray voltage); 350 °C (capillary temperature); 300 °C (vaporizer temperature).

The EA fraction (C 233.4 g) was passed through a silica gel column (200–400 mesh) using dichloromethane-MeOH (100:0, 15:1, 9:1, 8:2, 7:3, 1:1) to obtain seven sub-fractions (Fr. 1–7). Fr. 6 (32.3 g) was further separated by RP-18 column (open column, YMC, Kyoto, Japan) to get four fractions (Fr. 6a–d). Fr. 6b (5.2 g) was adsorbed on Sephadex LH-20 and then eluted with methanol-water (1:1) to obtain three sub-fractions (Fr. 6b1–b3). Fr. 6b1 (49 mg) was purified on prep-HPLC with ACN-water (5:1) as an eluent to yield compounds **6** (3.5 mg), **7** (4.2 mg) and **8** (8.1 mg). In a similar procedure, compounds **1** (6.1 mg), **3** (3.2 mg), and **5** (4.5 mg) were obtained from Fr. 7; and compounds **2** (3.1 mg) and **4** (2.9 mg) were obtained from Fr. 6d by HPLC. In addition, compound **9** was purified from fraction Fr. 6c (1.3 mg) on Sephadex LH-20 eluting with methanol-water (1:1).

The ^1^H NMR (600 MHz) and ^13^C NMR (125 MHz) spectra were recorded on a Bruker Avance 600. Column chromatography (CC) was performed on silica gel (200–400 mesh, Merck, Wuhan, China), Sephadex LH-20 (25–100 μm; Pharmacia Fine Chemical Co., Ltd. Uppsala, Sweden), and YMC Gel RP-18 (12 nm, S-150 um; YMC Co., Kyoto, Japan). Prep-HPLC analysis was performed on a LC6000 (Jiangsu Hanbang Co., Ltd. Jiangsu, China) instrument equipped with a Unitary C_18_ column (250 mm × 10 mm, 5 μm).

### 2.4. In vitro Antioxidant Assays of H. abyssinica

#### 2.4.1. 2,2-Diphenyl-1-Picrylhydrazyl (DPPH) Free Radical Scavenging Assay

The activities of the ethanol extract and its four solvent fractions were evaluated according to the previous method [29] with some modifications. Ascorbic acid (Vc) and BHT were used as positive controls in these assays. 200 µL DPPH-methanol (0.1 mM) was added to 3.8 mL of the adequately diluted samples (15.625–1000 mg/mL) and the mixtures were incubated at room temperature in darkness for 30 min. The absorbance was then measured at 517 nm with a multifunctional microplate reader (Tecan Infinite M200 PRO, TECAN, Männedorf, Switzerland). All of the samples and controls were analyzed in triplicate (*n* = 3). The final results were expressed as inhibition rate (%) and IC_50_ values. The results of antioxidant activity were calculated from the mean of three replicates (M ± SD). The DPPH free radical scavenging activity was calculated out as:

DPPH-free radical scavenging effect (%) = [(A_0_ − A)/A_0_] × 100%, (1)

where A_0_ and A are the absorbance value of the blank control and tested sample or positive control, respectively. The IC_50_ value represents the 50% inhibition ratio of DPPH activity.

#### 2.4.2. ABTS^+^ Radical Cation Scavenging Activity Assay

ABTS^+^ radical cation scavenging activity assay was carried out following the reported method [30] with a slight modification. Vc and Trolox were used as positive controls. In brief, the stock solution of ABTS^+^ (7 mM in H_2_O) was appropriately diluted with phosphate-buffered saline (pH 7.4) to get an absorbance of 0.700 ± 0.100 at a wavelength of 734 nm. Then, 200 μL of appropriately diluted samples with methanol were added to 4 mL of ABTS^+^ solution and shaken gently. The mixture was incubated in glass tubes for 6 min in darkness. ABTS^+^ scavenging activity was calculated as the scavenging effect in Equation (1) of Section 2.4.1. Results were expressed as the inhibition rate (%) and IC_50_ values.

#### 2.4.3. Ferric-Ion Reducing Antioxidant Power (FRAP) Assay 

The FRAP assay was performed in accordance with the method described previously by Benzie and Szeto [31]. The FRAP reagent was prepared by mixing 300 mM acetate buffer (pH 3.6), 10 mM TPTZ solution, and 20 mM FeCl_3_·6H_2_O at a ratio of 10:1:1 (*v/v/v*) and heated at 37 °C for 10 min. Appropriately diluted samples (0–80 µmol/L) were added to ultrapure water to reach 2 mL liquid solution, then fresh FRAP was added and incubated at 37 °C for 10 min. The absorbance of the mixture was recorded at 593 nm by triplicate tests (*n* = 3). Vc and BHT were used as the positive controls and FeSO_4_·7H_2_O was used to establish calibration curve. The FRAP activity assay was expressed as mg Fe^2+^/g of sample.

### 2.5. Determinations of Total Phenolic Content (TPC)

The TPC of the ethanol extract and the Hex, DCM, EA, and water fractions from *H. abyssinica* was determined by the Folin-Ciocalteu method, according to the determination of polyphenols in GB/T 8313-2008, and gallic acid was used as the standard. Adequate diluted sample (1–2 mg/mL) solution (1 mL) was mixed with 5 mL of Folin-Ciocalteu’s phenol reagent and incubated for 3 min. Then, 4 mL of sodium carbonate (15% *w/v*) was added to the mixture and incubated for 1 h in the dark. The absorbance of the samples was measured at 760 nm with UV/VIS spectrophotometer (UV-1100, MAPADA, Shanghai, China). The results were presented in the form of mg GAE (gallic acid equivalent)/g dry weight.

### 2.6. Determinations of Total Flavonoid Content (TFC)

The TFC of the ethanol extract and the Hex, DCM, EA, and water fractions from *H. abyssinica* were measured using the colorimetric assay [14,32]. Briefly, rutin was used as the standard and 2 mL of adequate diluted (0.5–1.5 mg/mL) sample solution, distilled water (3 mL), and 500 µL of NaNO_2_ (5% *w/v*) were put in a 10 mL conical tube. After incubation for 6 min, 500 µL of AlNO_3_ solution (10% *w/v*) was added. The sample-NaNO_2_-AlNO_3_ solution was incubated again for 6 min. Then, 4% NaOH solution (4 mL) was added to the sample-NaNO_2_-AlNO_3_ solution and incubated for 15 min. The UV/VIS spectrophotometer (UV-1100, MAPADA, Shanghai, China) was used to measure the resultant absorbance of the final mixture at 510 nm. The results were expressed as milligrams of rutin equivalent (RE) per gram of dry weight (mg RE/g dry weight). For each sample, the assay was repeated three times.

### 2.7. Anti-Proliferative Activity of H. abyssinica

The anti-proliferative activity was investigated according to the MTT assay, as previously described with minor modifications [33]. Liver cancer (HepG2), colon cancer (HT-29), and gastric tumor (SGC-7901) cell lines were obtained from the American Type Culture Collection (ATCC)and plated in 96-well microplates. All of the cells above were cultured in DMEM medium (GIBCO, Nanjing, China) and supplemented with 10% calf serum (SIJIQING, Nanjing, China). Cell suspension (100 μL) was added to each well of the 96-well cell culture plates and then the 96-well cell culture plates were cultured in 5% CO_2_ incubator (37 °C) for 24 h. Then, 100 μL of different concentrations of samples (9.375, 18.75, 37.5, 75, and 150 mg/mL) were added to the corresponding wells of the 96-well plate in triplicates and the negative control group was also set up. After 72 h incubation, 20 μL of MTT (5 mg/mL) was added to each well at 37 °C and incubated for 4 h. Then, 150 mL DMSO was added to each well after the supernatant was discarded. After shaking for 15 min, the absorbance of the sample was measured at 590 nm with a microplate reader (Tecan Infinite M200 PRO, TECAN, Männedorf, Switzerland). Graphpad Prism software 6.0 (GraphPad Software Inc., San Diego, CA, USA) was used to calculate the IC_50_ values.

### 2.8. Statistical Analysis

Each sample testing was repeated three times. All results were presented as means ± standard deviation. Experimental data were subjected to Origin 8.0 (OriginLab Corporation, Northampton, MA, USA), Graphpad Prism software 6.0, SPSS 19.0 (SPSS Inc., Chicago, IL, USA) and Chemdraw 14.0 (CambridgeSoft Corp., Cambridge, MA, USA). For all of the analyses, the differences were considered statistically significant at the *p* < 0.01 level.

## 3. Results and Discussion

### 3.1. In Vitro Antioxidant Activity of H. abyssinica

**Figure 1:**
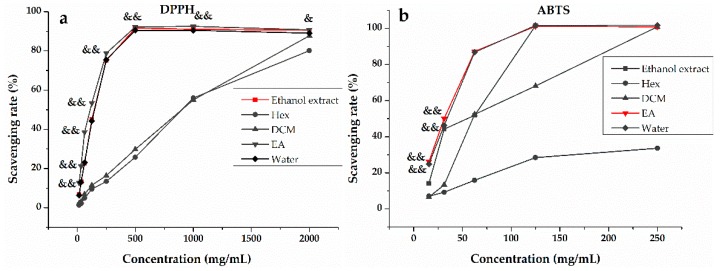
The scavenging rate (%) of the ethanol extract, *n*-hexane (Hex), dichloromethane (DCM), ethyl acetate (EA), and water fractions of *H. abyssinica*: (**a**) the radical scavenging rate (%) of 2,2-diphenyl-1-picrylhydrazyl (DPPH) and (**b**) the radical scavenging rate (%) of 2,2′-azino-bis(3-ethylbenzthiazoline-6-sulfonic acid (ABTS). All of the values in the figure are expressed as means (%) and SD of triplicated experiments. ^&^ *p* < 0.05, ^&&^ *p* < 0.01, compared with the ethanol extract.

**Figure 2:**
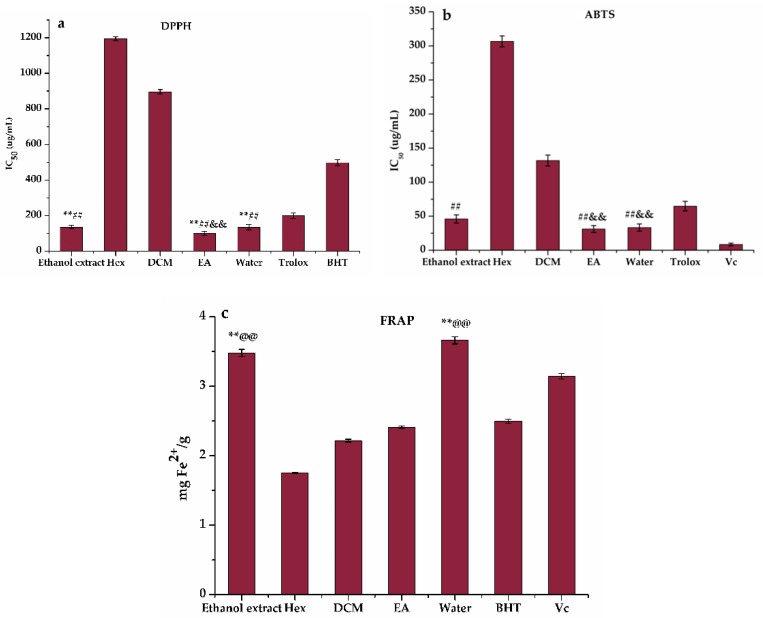
Antioxidant activity of the ethanol extract, *n*-hexane (Hex), dichloromethane (DCM), ethyl acetate (EA) and water fractions of *H. abyssinica*: (**a**) The IC_50_ value of 2,2-diphenyl-1-picrylhydrazyl (DPPH) radical scavenging assay, (**b**) The IC_50_ value of 2,2′-azino-bis(3-ethylbenzthiazoline-6-sulfonic acid (ABTS) radical scavenging assay, and (**c**) ferric-ion reducing antioxidant power (FRAP) assay. ^##^ *p* < 0.01, ** *p* < 0.01, ^@@^ *p* < 0.01, and ^&&^ *p* < 0.01, as compared with the positive controls Trolox, BHT, Vc, and ethanol extract, respectively.

To fully evaluate antioxidant activity, a series of methods were used in parallel due to the different scavenging modes of ROS and the complexity of natural phytochemicals [34]. In this work, the three most representative assays (DPPH, ABTS, FRAP) were implemented to assess and compare the antioxidant potential of the ethanol extract and its Hex, DCM, EA, and water fractions of *H. abyssinica*. Figure 1 shows that the ethanol extract, together with its Hex, DCM, EA, and water fractions, had some definite scavenging effect on DPPH (Figure 1a) and ABTS (Figure 1b) and the scavenging rate was dose-dependent. As shown in Figure 2, the EA fraction showed significant activities in DPPH and ABTS radical scavenging assays (Figure 2a,b) with IC_50_ values of 99.700 ± 0.013 g/mL and 31.200 ± 0.001 g/mL, as compared to the positive controls (Trolox with IC_50_ = 198.680 ± 0.010 and 64.760 ± 0.003 g/mL, respectively). Moreover, the results of FRAP (Figure 2c) revealed that the reduction ability was the strongest in the ethanol extract with 3.478 mg Fe^2+^/g, followed by the EA and water fractions. Despite the differences in antioxidant capacity of the ethanol extract and the Hex, DCM, EA, and water fractions observed in the three methods (DPPH, ABST, and FRAP), the EA fraction integrally exhibited better antioxidant potential than the other three fractions and the ethanol extract of *H. abyssinica*. Hence, the phytochemical study of EA fraction was then conducted to reveal the specific compounds with the best antioxidant activity.

### 3.2. Total Phenolic and Flavonoid Contents of H. abyssinica

**Figure 3:**
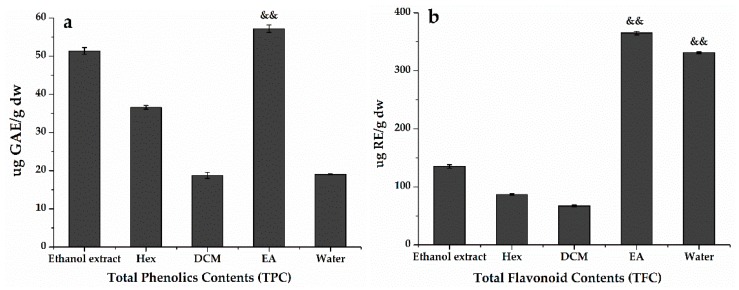
(**a**)Total phenolics contents (TPC) and (**b**) total flavonoids contents (TFC) of *H. abyssinica*. GAE/g dw: gallic acid equivalent per gram of dry weight; RE/g dw: rutin equivalent per gram of dry weight. ^&&^ *p* < 0.01, as compared with the ethanol extract.

In order to further explore the potentially active compounds in *H. abyssinica* with antioxidant activity, the TPC in the ethanol extract and Hex, DCM, EA, and water fractions were determined using the equation (*y* = 0.0096*x* + 0.1279, *R*² = 0.9977) obtained by calibration curves and the TFC was *y* = 0.0025*x* + 0.1005, *R*² = 0.9994. The results in Figure 3 showed that *H. abyssinica* had high flavonoids and polyphenols. The TPC assay (Figure 3a) revealed that the greatest accumulation of phenolics occurred in EA (57.193 ± 0.001 μg GAE/g), followed by the ethanol extract (51.347 ± 0.001 μg GAE/g) and Hex (36.573 ± 0.001 μg GAE/g). Similarly, EA contained the most abundant flavonoids (365.091 ± 0.001 μg RE/g). This level was 5.4-times higher than that in DCM (67.190 ± 0.001 μg RE/g) and 4.2-times higher than that in Hex (86.733 ± 0.001 μg RE/g) (Figure 3b). The content of flavonoids and polyphenols had a certain degree of positive correlation with the antioxidant effect. Hence, it could be assumed that TPC and TFC in the *H. abyssinica* extract might be related to the ability of scavenging free radicals.

### 3.3. Anti-Proliferative Activity of H. abyssinica

**Figure 4:**
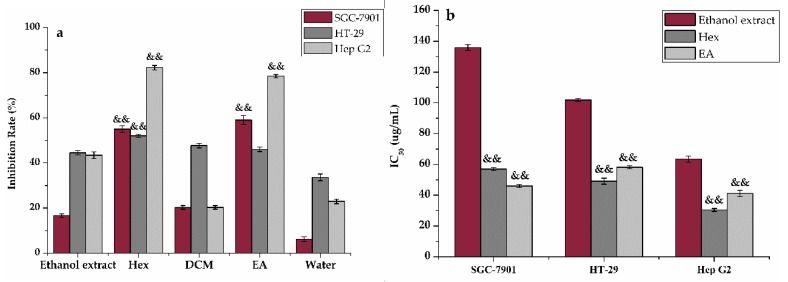
Anti-proliferative activity of the ethanol extract, Hex, DCM, EA, and water fractions of *H. abyssinica*: (**a**) The inhibition ratio of SGC-7901, HT-29, and HepG2 cells treated by the ethanol extract, Hex, DCM, EA, and water fractions of *H. abyssinica*; and (**b**) The IC_50_ value of the ethanol extract, Hex, and EA. All of the values in the figure are expressed as means (%) and standard deviation of triplicate experiments. ^&&^ *p* < 0.01, as compared with the ethanol extract.

Anti-proliferative activity was strongly associated with antioxidant activity because antioxidants can effectively restrain the formation and occurrence of cancers caused by oxidative stress, which may lead to metabolic malfunctions and oxidative damage of biological macromolecules [15]. Extensive investigations revealed that regulation of the level of ROS could reduce the incidence of cancers and could also be useful in the treatment of cancers. Paul et al. [35] identified a SIRT3 pathway by which the survival and proliferation of tumor cells were suppressed through ROS regulation. Li et al. [36] investigated the effects of dalbinol, which could induce apoptosis of human colon cancer cells through the ROS/Dvl/GSK-3β/β-catenin pathway. Hence, it was necessary to conduct the anti-proliferative assay to assess the potential relationship between antioxidant and anti-proliferative activity of the ethanol extract and the Hex, DCM, EA, and water fractions of the roots of *H. abyssinica* with the MTT method. Figure 4 shows that the Hex and EA fractions of the plant possessed better anti-proliferative activities with a higher inhibition rate (50 μg/mL) to Hep G2, SGC-7901 and HT-29 cell lines than the other three samples. Previous reports have shown that *H. abyssinica* had a certain inhibitory effect on leukemia, but the potential anti-proliferative activities of *H. abyssinica* were rarely reported to date. Figure 4 showed that *H. abyssinica* had potential anti-proliferative activities. Hence, it further proved that the main active substances with antioxidant and anti-proliferative activities could be enriched in the EA fraction.

### 3.4. Potential Antioxidants and Anti-Proliferative Compounds

**Figure 5:**
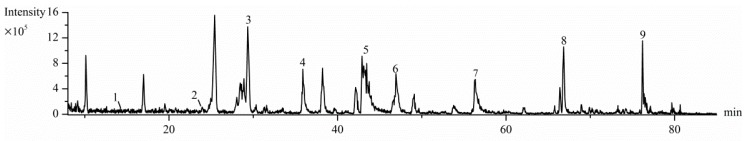
The LC-MS base peak chromatogram (BPC) of *H. abyssinica* (EA fraction) in the negative ion mode.

**Figure 6:**
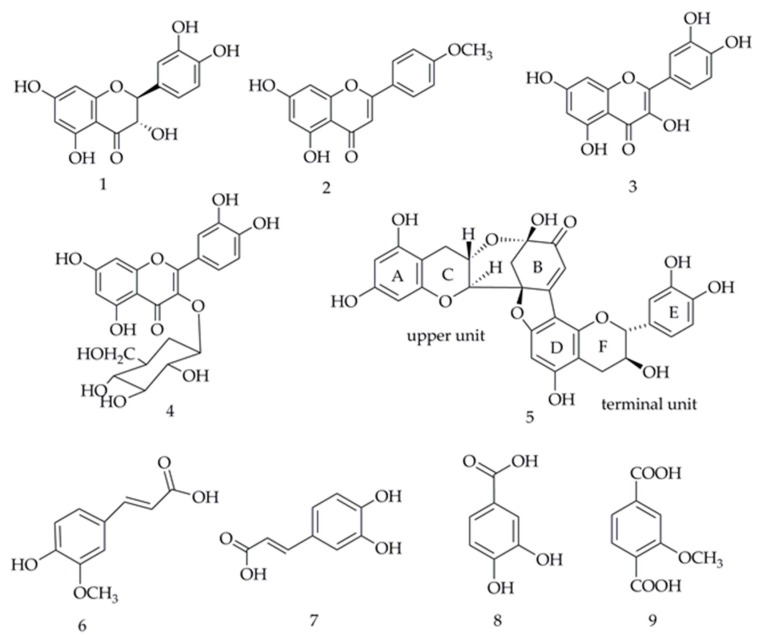
The nine compounds as potential antioxidants from *H. abyssinica*.

Phenols and flavones are usually suggested to be the major compounds with antioxidant capacity in plants [37]. The results of the TPC and TFC from *H. abyssinica* further showed that phenols and flavones were the main substances displaying antioxidant activity. Meanwhile, the EA fraction of *H. abyssinica* showed stronger antioxidant activity when compared with the other four samples, as seen in on Figure 2. At present, there is no report on the systematic separation of the EA fraction and only a few phenols and flavone compounds were reported from *H. abyssinica*. In this study, the EA fraction was firstly subjected to HPLC-UV/ESI-MS/MS analysis (Figure 5 and Table 1), then the specific antioxidants compounds were isolated and identified. As shown in Figure 6, five flavonoids and four phenolics, namely dihydroquercetin (**1**), acacetin (**2**), quercetin (**3**), isoquercitin (**4**), dehydrodicatechin A (**5**), trans-ferulic acid (**6**), caffeic acid (**7**), 3,4-dihydroxybenzoic acid (**8**), and 2-methoxyterephthalic acid (**9**), were separated from the EA fraction of *H. abyssinica,*. Their structures were elucidated based on NMR and other spectroscopic methods. Compounds **1**–**3**, **5**–**7**, and **9** were identified for the first time from *H. abyssinica* as well as from the Hagen genera.

The compound **5** (yellow powder) was obtained as the oxidation product of (+)-catechin and the spectroscopic data of NMR (^1^H, ^13^C, HMBC, COSY, NOESY) were consistent with that of dehydrodicatechin A [47,48], as shown in Table 2. The carbon resonances (*δ*c: 27 to 84) in the heterocyclic C and F rings presented as twin peaks with comparable abundance, which implied that compound **5** had a pair of catechin carbon signals when combined with the ^13^C-NMR and distortionless enhancement by polarization transfer spectrum (DEPT-NMR) [38]. In addition, the carbonyl signal at *δ*_C_: 194.13, methylene group at *δ*_C_: 45.96, two quaternary carbons at *δ*_C_: 89.86 and 95.28, and two olefin carbons at *δ*_C_: 112.85 and 164.37 from the B ring were the major differences from the un-substituted aromatic carbon of the E ring. Meanwhile, the F-ring signals *δ*_H_: 2.60 (1H, dd, *J* = 7.5, 16.5 Hz, H-4axF), 2.85 (1H, dd, *J* = 5.0, 16.5 Hz, H-4exF), 4.11 (1H, m, H-3F), and 4.93 (1H, d, *J* = 6.5 Hz, H-2F) in the ^1^H-NMR spectrum were similar to C-ring signals at *δ*_H_: 2.50 (1H, m, H-4axC), 2.94 (1H, m, H-4exC), 3.98 (1H, m, H-2C), and 3.98 (1H, m, H-3C). In addition, the E-ring aromatic signals *δ*_H_: 6.84 (1H, d, J = 2.0 Hz, H-2′E), 6.74 (1H, dd, *J* = 2.0, 8.0 Hz, H-6′E), and 6.79 (1H, dd, *J* = 8.0 Hz, H-5′E) showed obvious differences from B-ring signals at *δ*_H_: 6.43 (1H, s, H-5′B), 2.67 (1H, m, H-2′B), and 2.50 (1H, m, H-2′B). The two methenyl group signals at *δ*_H_: 5.54 (1H, d, *J* = 2.0 Hz, H-8A) and 5.90 (1H, d, *J* = 2.0 Hz, H-6A) belonged to the A-ring, while the methenyl group signal at *δ*_H_: 6.12 (1H, s, H-6D) belonged to the D-ring. The reference stereochemistry of compound **5** was also confirmed by the circular dichroism (CD) spectrum and the nuclear overhauser effect spectroscopy (NOE) experiment and the values of *δ*_H_ and *δ*_C_ were recorded by reference (Methanol-*d*_4_: *δ*_H_ 3.31/*δ*_C_ 49.0), as shown in Table 2.

### 3.5. The Structural Features and the Antioxidant and Anti-Proliferative Activities of Potential Active Compounds

The core structure of flavones is two benzene rings, A and B, linked by a heterocyclic pyrane C ring (C6-C3-C6), and the functional groups of flavones are hydroxy, carbonyl, and conjugated double bonds [49,50]. The antioxidant and anti-proliferative effects of flavones are closely related to their structures, such as (1) the difference of various flavones mother nucleus, (2) the position, number, and degree of hydroxyls, (3) C-2,3 double bonds, and (4) the other modifications [51,52]. Habtemariam et al. [53] found that eriodictyol with 3′,4′-OH showed the strongest protective effect on L-929 cells from tumor necrosis factor (TNF)-induced cell death, while hesperidin (with 3’-OH but without 4’-OH) had no protective effect. It indicated that the flavones with 3’,4’-OH had strong antioxidant activity, like dihydroquercetin and quercetin (compounds **1** and **3**) [54,55,56,57]. Dihydroquercetin (compound **1**) as well-known antioxidants protect DNA from oxidative damage and against oxidative stress by stimulating the expression of HO-1 and NQO1 through the Nrf2-dependent antioxidant pathway [58]. Saelooom Lee et al. found that IM3829 is a promising radio-sensitizer to human lung cancer cells by blocking the antioxidant responses (Nrf2-dependent), which further demonstrates that the regulation of the anti-oxidation pathway can effectively prevent the occurrence and development of cancer [59]. Gulati et al. found quercetin (compound **3**) as an anti-oxidative flavonoid showed anti-proliferative effect on cancer cells by inhibiting the PI3K-Akt/PKB pathway, the major target of antioxidants [60] and Hsu et al. found quercetin inhibited the proliferation of Hep G2 by increasing the expression of p53 and p21/WAF1, leading to cell cycle stagnation and apoptosis of Hep G2 [61].

The glycosylation of C-3 hydroxyl led to a mild effect on the antioxidant and anti-proliferative activity of flavones. On the contrary, the isoquercitin (compound **4**) showed significant antioxidant activity and the capacity to reduce glioblastoma cell proliferation and change β-catenin cellular localization, which may be due to the 3’,4’-OH and C-2,3 double bonds in terms of the structure-activity relationship [51,62,63,64]. Previous studies have suggested that ring A is not easily oxidized, so it does not directly participate in antioxidant reactions. However, recent studies have shown that the A ring is also involved in antioxidant activity and the presence of the C-5 and C-7 hydroxyl groups on the A ring are beneficial to antioxidant activity [65]. Lopez-Posadas et al. found that C-2,3 double bonds and 3′,4′-OH could enhance the anti-proliferative activity of flavones [66]. So the antioxidant and anti-proliferation activity of acacetin (compound **2**) could most likely be due to the present of C-5, C-7 hydroxyl groups in the A ring and 3′,4′-OH, 3-OH and the C-2,3 double bond [47,67]. Meanwhile, Shen et al. [68] discovered acacetin could inhibit the human prostate cancer DU145 cells by regulating and controlling the antioxidants-related signaling pathways-p38 MAPK.

Up to now, diflavones with strong antioxidant and anti-proliferative activity have been reported [69,70], for example, compound **5** with ortho-hydroxyl structures showed significant antioxidant activity and protective effects on anoxia-induced injury in the cultivation of ECV304 or PC12 cells with the IC_50_ at 50 µg/mL [47,48,71]. There is an important relationship between the antioxidant activity of polyphenol and its hydroxyl groups [72], where the higher the number of phenolic acid hydroxyl groups, the stronger the antioxidant activity of polyphenol. Phenolic acids were divided into several types, such as cinnamic acid, benzoic acid, and ethyl acid. Chen et al. [73] found that the phenolic acid type was an important factor affecting antioxidant activity, for example, the type of cinnamic acid (trans-ferulic acid, caffeic acid) had stronger antioxidant activity than benzoic acid (3,4-dihydroxybenzoic acid, 2-methoxyterephthalic acid). 3,4-dihydroxybenzoic acid (compound **8**) enriched in anti-oxidative foods, like fruits and vegetables, could prevent the growth of carcinogenesis in vivo through controlling the phosphorylation and activation of JNK and p38 MAPK signal pathway [74]. In addition, synergistic antioxidant effects of flavones and phenolic acids further indicated that total flavonoids and total polyphenols could be the main active components of antioxidants [75,76]. At present, the anticancer properties of phenolic acids are increasingly studied and some have shown potential anti-proliferative activities [64].

**Figure 7:**
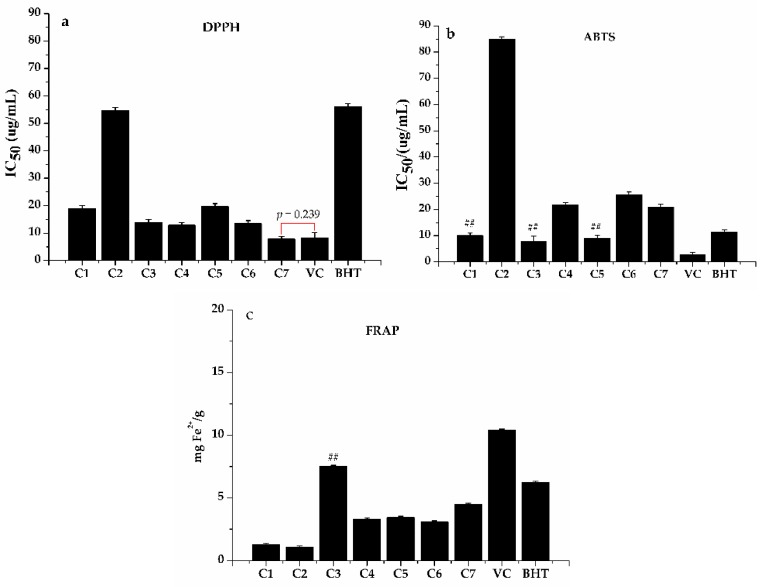
The antioxidant activity of compounds from the EA fraction, C1 = dihydroquercetin, C2 = acacetin, C3 = quercetin, C4 = isoquercitin, C5 = dehydrodicatechin A, C6 = trans-ferulic acid, and C7 = caffeic acid. (**a**) The IC_50_ value of DPPH radical scavenging assay, (**b**) The IC_50_ value of ABST radical scavenging assay, and (**c**) ferric-ion reducing antioxidant power (FRAP) assay. ^##^ *p* < 0.01 compared with the positive control of BHT.

**Figure 8:**
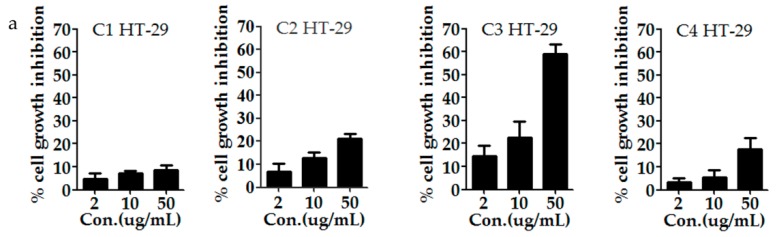

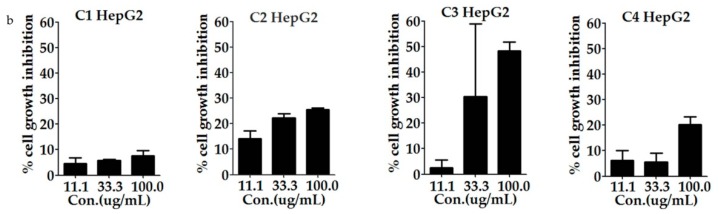
Anti-proliferative activity of some compounds from the EA fraction of *H. abyssinica*, C1 = dihydroquercetin, C2 = acacetin, C3 = quercetin, and C4 = isoquercitin. (**a**) The inhibition ratio of HT-29 treated by compounds C1–C4 of *H. abyssinica*. (**b**) The inhibition ratio of HepG2 treated by compounds C1–C4 of *H. abyssinica*. All of the values in the figure are expressed as means (%) and standard deviation of triplicated experiments.

The antioxidant and anti-proliferative activities of those compounds from the active fraction tested were also evaluated with the same methods in Section 2.4 (DPPH, ABTS, FRAP) and Section 2.6 (MTT) in order to further verify the relationships between the activity and structure of those compounds. The results are shown in Figure 7 and Figure 8. The compound **7**, with cinnamic acid structure, showed the strongest DPPH scavenging ability with an IC_50_ value of 7.858 ± 0.31 µg/mL, which was even better than the positive control (Vc, IC_50_ 8.27 ± 0.11 µg/mL) and the result agreed with Gong et al. [77]. Acacetin had the lowest DPPH scavenging ability with an IC_50_ value of 54.749 ± 1.2 µg/mL. Meanwhile, compound **3**, **4**, and **6** had similar clearance rates to DPPH (13.984 ± 0.21, 12.913 ± 0.11, and 13.548 ± 0.19 µg/mL, respectively). As shown in Figure 7b, compound **1**, **3**, and **5** showed stronger antioxidant activity than that of other compounds in ABST^+^ scavenging abilities with IC_50_ values of 9.978 ± 0.17, 7.885 ± 0.23, and 9.041 ± 0.77 µg/mL, respectively. As seen in Figure 7c, compound **3** (7.492 mg Fe^2+^/g) exhibited the strongest reducing power among all the compounds tested, which was even stronger than BHT (6.228 mg Fe^2+^/g). Others were weaker than BHT and Vc (6.228 mg Fe^2+^/g and 10.388 mg Fe^2+^/g). Acacetin exhibited the lowest reducing power with an IC_50_ of 1.076 mg Fe^2+^/g, as shown in Figure 7c. In a word, the compounds with 3′,4′-OH and the C-2,3 double bones, like **1**, **3**, and **4**, showed strong oxidative resistance in DPPH, ABST, and FRAP assays. Meanwhile, the anti-proliferation activity, shown in Figure 8a,b, showed that dihydroquercetin, acacetin, quercetin, and isoquercitin had concentration-dependent inhibitory effects on HT-29 and Hep G2 cells to some extent and compound **3** showed relatively stronger inhibitory activity compared with the other three.

## 4. Conclusions

*H. abyssinica* can be used as a potential medicinal resource for chemoprevention partly due to those potential active compounds from its roots with good antioxidant and anti-proliferative properties. In this study, the antioxidant activities of different extracts and subsequent fractions were measured with DPPH, ABTS, and FRAP methods, along with the anti-proliferative activity, tested by MTT. For the first time, the correlations among antioxidant, anti-proliferative activities, and phytochemical compounds of *H. abyssinica* were demonstrated. The results showed that the concentration-dependent antioxidant properties of the EA fraction had the best antioxidant activity because of the presence of higher levels of phenylpropanoids, which are the largest group of plant secondary metabolites and are naturally occurring antioxidants. The EA fraction was also found to possess significant anti-proliferative properties, with the highest TPC and TFC contents. These facts can partly explain its greater anti-proliferative and antioxidant effects than that of the other fractions. More importantly, nine compounds from the EA fraction, including 4 phenolic compounds and 5 flavonoids, were then isolated and identified to be the main active components, contributing to both anti-oxidative and anti-proliferative activities. It was then revealed that caffeic acid (compound **7**) showed stronger DPPH scavenging ability (IC_50_ 7.858 ± 0.31 µg/mL) when compared with the positive control (Vc, IC_50_ 8.27 ± 0.11 µg/mL) and quercetin (compound **3**) showed relatively stronger antioxidant activities than the other six compounds in the ABTS and FRAP assay. Meanwhile, the quercetin showed the strongest concentration-dependent anti-proliferative activities on the HT-29 and HepG2 cells among all of the compounds tested.

## Figures and Tables

**Table 1:** The LC-MS/MS data of nine compounds in the EA fraction of *H. abyssinica.*

Compound	Peak No.	Rt/min	[M – H]^−^	MS/MS Spectrum	Identification	Ref.
1	6	46.47	303.24	285.13, 241.10, 217.08, 199.16, 174.68, 125.18	dihydroquercetin	[38]
2	9	76.12	283.23	268.06, 240.03, 239.03, 211.03, 212.04, 151.00, 117.03, 107.01	acacetin	[39]
3	8	66.76	301.50	179.11,151.07	quercetin	[40]
4	5	42.90	463.23	301.24, 300.14	isoquercitin	[41]
5	7	56.62	575.06	445.00, 423.00, 394.00, 271.00, 243.00, 229.00, 137.00, 125.00	dehydrodicatechin A	[42]
6	4	35.78	193.10	178.10,149.12,134.08	*trans*-ferulic acid	[43]
7	2	24.15	179.48	135.21	caffeic acid	[44]
8	1	14.55	153.11	109.02	3, 4-dihydroxybenzoic acid	[45]
9	3	29.22	195.16	180.23, 136.11	2-methoxyterephthalic acid	[46]

**Table 2:** 600 MHz ^1^H-NMR and 150 MHz ^13^C-NMR data of compound **5** in Methanol-*d*_4_.

	^1^H-NMR	^1^H-^1^H COSY^a^	^13^C-NMR	HMBC^b^ (H→C)
2C	3.98 (1H, m)	4C-H	79.5	C-3C, 4C, 6′B
3C	3.98 (1H, m)	3C-H	66.9	C-2C, 4C, 1′B
4C	Ha: 2.50 (1H, m, 4axC) Hb: 2.94 (1H, m, 4exC)	3C-H	27.8	C-2C, 3C, 4aA, 5A
4aA	-		100.5	-
5A	-		156.4	-
6A	5.54 (1H, d, *J* = 2.0 Hz)	8A	97.1	C-4aA, 5A, 7A, 8A
7A	-		157.7	-
8A	5.90 (1H, d, *J* = 2.0 Hz)	6A	95.8	C-4aA, 6A, 7A, 8aA
8aA	-		158.0	-
1′B	-		89.9	-
2′B	Ha: 2.50 (1H, m) Hb: 2.67 (1H, m)	3′B-H	45.9	C-2C, 1′B, 3′B, 6′B
3′B	-	2′B-H, 4′B-H	95.3	-
4′B	-	3′B-H	194.1	-
5′B	6.43 (1H, s)	3′B-H	112.9	C-2′B, 1′B, 3′B, 6′B
6′B	-		164.4	-
2F	4.93 (1H, d, *J* = 6.5 Hz)		83.5	C-3F, 4F, 1′E, 2′E, 6′E, 8aD
3F	4.11 (1H, m)		67.9	C-4aD
4F	Ha: 2.60 (1H, dd, *J* = 7.5, 16.5 Hz, 4axF) Hb: 2.85 (1H, dd, *J* = 5.0, 16.5 Hz, 4exF)		28.3	C-2F, 3F, 4aD, 5D, 8aD
4aD	-		103.9	
5D	-		166.2	
6D	6.16 (1H, s)		90.9	C-5D, 8D
7D	-		168.1	
8D	-		105.6	
8aD	-		155.1	
1′E	-		131.3	
2′E	6.84 (1H, d, *J* = 2.0 Hz, H-2′E)	5′E	114.8	C-2F, 4′E, 6′E
3′E	-		146.5	
4′E	-		146.6	
5′E	6.79 (1H, dd, *J* = 8.0 Hz, H-)	2′E, 6′E	116.3	C-1′E, 3′E
6′E	6.74 (1H, dd, *J* = 2.0, 8.0 Hz)	5′E	119.7	C-2F, 2′E, 4′E

^a^: ^1^H-^1^H Correlation Spectroscopy; ^b^: Heteronuclear Multiple Bond Correlation; “-”: No associated carbon and hydrogen signals.
